# Hydrogen Peroxide Generation of Copper/Ascorbate Formulations on Cotton: Effect on Antibacterial and Fibroblast Activity for Wound Healing Application

**DOI:** 10.3390/molecules23092399

**Published:** 2018-09-19

**Authors:** J. Vincent Edwards, Nicolette T. Prevost, Michael Santiago, Terri von Hoven, Brian D. Condon, Huzaifah Qureshi, Dorne R. Yager

**Affiliations:** 1 Southern Regional Research Center, USDA-ARS, New Orleans, LA 70120, USA; nicolette.prevost@ars.usda.gov (N.T.P.); michael.santiago@ars.usda.gov (M.S.); tm.vonhoven@ars.usda.gov (T.v.H.); brian.condon@ars.usda.gov (B.D.C.); 2 Plastic and Reconstructive Surgery, Virginia Commonwealth University, Richmond, VA 23111, USA; qureshihs@mymail.vcu.edu (H.Q.); dorne.yager@vcuhealth.org (D.R.Y.)

**Keywords:** cotton, hydrogen peroxide, wound dressings, copper, ascorbate, nanoparticles, antibacterial

## Abstract

Greige cotton (unbleached cotton) is an intact plant fiber that retains much of the outer cotton fiber layers. These layers contain pectin, peroxidases, and trace metals that are associated with hydrogen peroxide (H_2_O_2_) generation during cotton fiber development. When greige cotton is subjected to a nonwoven hydroentanglement process, components of the outer cotton fiber layers are retained. When hydrated, this fabric can generate H_2_O_2_ (5–50 micromolar). This range has been characterized as inducing accelerated wound healing associated with enhanced cell signaling and the proliferation of cells vital to wound restoration. On the other hand, H_2_O_2_ levels above 50 micromolar have been associated with bacteriostatic activity. Here, we report the preparation and hydrogen peroxide activity of copper/ascorbate formulations, both as adsorbed and in situ synthesized analogs on cotton. The cooper/ascorbate-cotton formulations were designed with the goal of modulating hydrogen peroxide levels within functional ranges beneficial to wound healing. The cotton/copper formulation analogs were prepared on nonwoven unbleached cotton and characterized with cotton impregnation titers of 3–14 mg copper per gram of cotton. The copper/ascorbate cotton analog formulations were characterized spectroscopically, and the copper titer was quantified with ICP analysis and probed for peroxide production through assessment with Amplex Red. All analogs demonstrated antibacterial activity. Notably, the treatment of unbleached cotton with low levels of ascorbate (~2 mg/g cotton) resulted in a 99 percent reduction in *Klebsiella pneumoniae* and *Staphylococcus aureus*. In situ synthesized copper/ascorbate nanoparticles retained activity and did not leach out upon prolonged suspension in an aqueous environment. An assessment of H_2_O_2_ effects on fibroblast proliferation are discussed in light of the copper/cotton analogs and wound healing.

## 1. Introduction

Largely because cotton-based dressings possess a good absorptive capacity and are inexpensive, they are one of the most widely employed materials for the treatment of exudative wounds [1]. An attractive goal would be to impart additional functionality to cotton-based dressings and yet maintain their other desirable properties.

At low concentrations, reactive oxygen species (ROS) may serve as signaling messengers in the cell and regulate numerous signal transduction and gene expression processes [2]. During wound repair, ROS are expressed by both phagocytic and non-phagocytic cells [3]. Roy et al., demonstrated that low concentrations of H_2_O_2_ are present in healing wounds and that the removal of H_2_O_2_ inhibits healing [4]. Hydrogen peroxide generation that either enhances cell proliferation or promotes antimicrobial activity has been a subject of recent mechanistic and therapeutic interest [4,5,6,7]. Therapeutic chronic wound dressings with functionalities targeted to low-level hydrogen peroxide generation (5–50 µM) have been of interest in dressing design for the promotion of cell proliferation since Schmidt et al. [8] first demonstrated low-level hydrogen peroxide based on compositional functionality in semi-occlusive dressings. Moreover, recently, approaches to modulate H_2_O_2_ levels with bio-electric dressings have been reported [9,10,11]. The application of hydrogen peroxide as an antibacterial has been known for over a century, yet has been re-visited recently in the context of biofilms [12]. The recent interest in the use of honey to treat wounds may be partly the result of its production of low levels of hydrogen peroxide [13,14].

Notably, the differences in wound healing upon application of the two functional levels of H_2_O_2_ have been characterized in an in vivo model [4], and characterized based on clinical dosage for the potential to use an economical naturally occurring material source as a chronic wound dressing. Reports on the positive efficacy of H_2_O_2_ delivery in vivo merit the development of such promising materials [4]. Thus, in this regard, we have endeavored to understand modification of the unbleached cotton fiber to produce sustained levels of hydrogen peroxide.

It has recently been determined that greige cotton generates low levels of H_2_O_2_ (5–50 micromolar). However, the mechanism of action of hydrogen peroxide production in the cotton fiber may be considered multifactorial, i.e., trace metals [15,16], polyphenols [15,17], peroxidase [17], super oxidase dismutase [18,19], and pectin [17,20], which are found in both brown and white cotton varieties, have been correlated with activity [15]. This study examines hydrogen peroxide production from unbleached cotton based on formularies prepared by simple modifications adoptable for dressing development. The principle objectives were to: (1) determine whether the addition of copper/ascorbate (Asc) formulations to greige cotton can augment hydrogen peroxide generation; (2) assess the relative roles of copper and ascorbate formulations in generating hydrogen peroxide production; and (3) examine the effects of these cotton formulations on cell proliferation.

## 2. Results

### 2.1. Formation and Characterization of Copper Complexes on Cotton

Both adsorption and in situ synthesis of copper micro- and nano-particles were performed by way of pad-dry and in-situ nanoparticle synthesis on nonwoven hydroentangled unbleached cotton [21,22,23]. The incorporation of copper on the cotton fabrics was quantified and found to range from 3–14 mg/g (Table 1).

**Figure 1:**
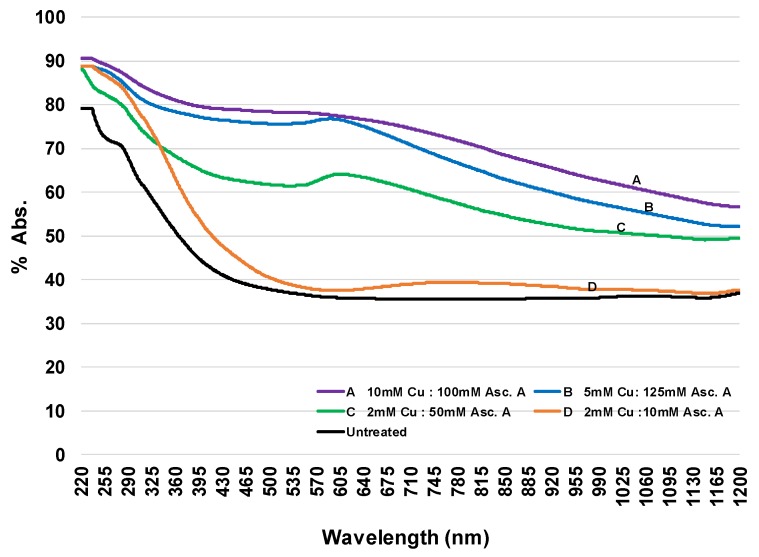
Solid UV/Vis spectra of hydroentangled 100% greige cotton with in situ copper nanoparticles (**A**–**C**) and adsorbed (**D**). Spectra (**A**–**C**) were pretreated with NaOH, treated with different concentrations of copper chloride solution, and reduced with ascorbic acid. Spectrum D was pad-dry treated with copper chloride in an ascorbic acid solution. The copper nanoparticle (CuNP) fabric samples exhibited a characteristic localized surface plasmon resonance peak at ~580 mn and the adsorbed sample copper oxide at ~800 nm.

To characterize the incorporation of copper nanoparticles on cotton, surface plasmon resonance detection was measured with solid state ultraviolet-visible spectroscopy, as shown in Figure 1. The use of this approach is based on the display by transition metal nanoparticles of characteristic absorption peaks [24] in the UV-Visible region. For example, copper nanoparticles incorporated into cotton with the in situ synthesis give a plasmon peak at 570–580 nm in a dose-responsive manner. Incorporation of the copper oxide in the cotton is ascertained by the presence of a characteristic peak around 800 nm (spectrum d) [25].

### 2.2. Production of Hydrogen Peroxide Levels from Nonwoven Greige Cotton

**Figure 2:**
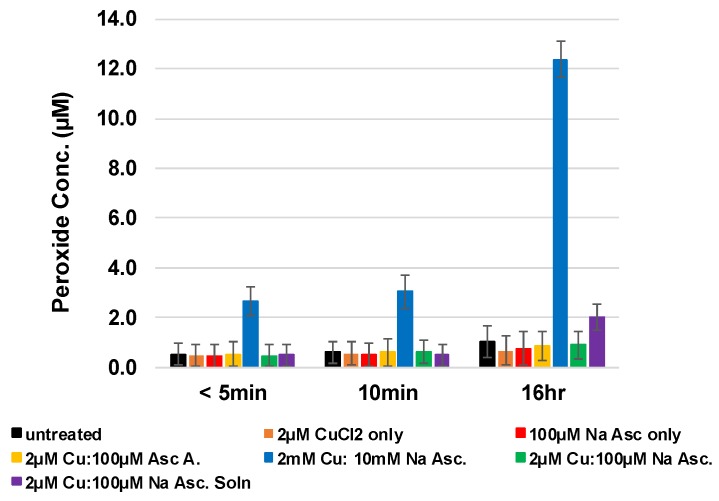
Nonwoven HE 100% greige cotton fabric Pad-Dry treated with copper chloride in sodium ascorbate solution concentrations listed in Table 2 compared to previous fabric adsorbed concentration as a control.

As shown in Figure 2, millimolar Cu/NaAsc formulations adsorbed by nonwoven unbleached cotton were effective in producing hydrogen peroxide. However, as shown in Table 1, an analysis of a range of ascorbate and copper concentrations performed independent of the cotton was examined to assess the production of hydrogen peroxide in solution. Thus, copper/ascorbate solutions prepared and tested independently at a concentration range one order of magnitude lower than solutions applied to the cotton-based formulations exhibit comparable hydrogen peroxide generation to that found at a millimolar level Cu/Asc-cotton formulation. It is important to note that as shown in Table 1, the cotton-copper-ascorbate formulations resulted in a copper adsorption of 2–14 mg/g fabric. 

Conversely, micromolar Cu/Asc in solution generates low-level hydrogen peroxide (median hydrogen peroxide production of 25 micromolar). Threshold levels associated with enhanced cell proliferation are achieved with copper/ascorbate adhered to the unbleached cotton in the range of 2–14 mg Cu/g fabric.

### 2.3. Assessment of Hydrogen Peroxide Levels from Adsorbed versus In Situ Adhered Copper

**Figure 3:**
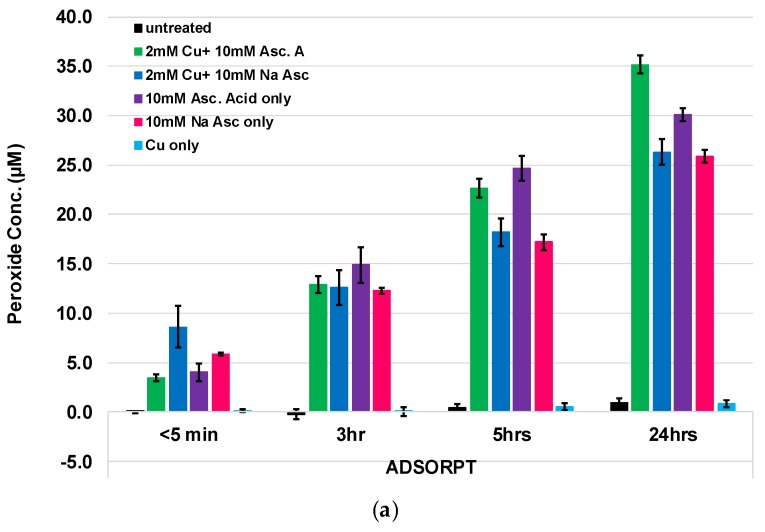

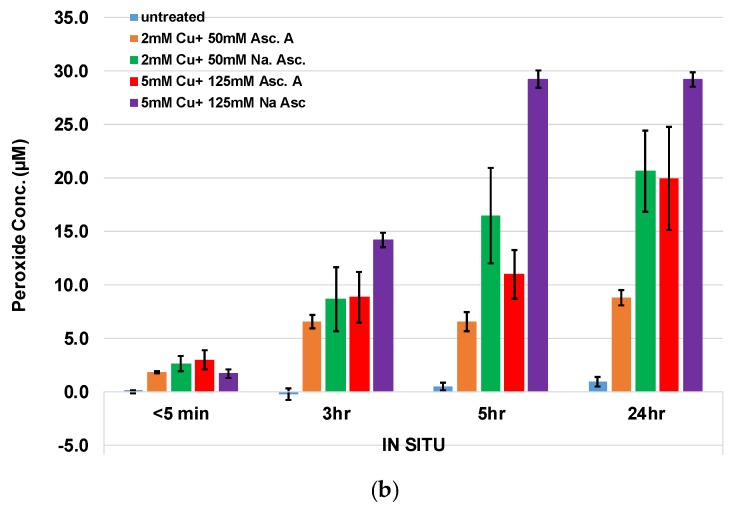
Peroxide generation of hydroentangled nonwoven greige cotton fabric enhanced using different methods, i.e., (**a**) surface adsorption and (**b**) in situ formation of copper particles determined by the Amplex Red assay, with the [AR] and [HRP] final concentration totaling 200 µM and 0.05 U/mL, respectively.

As shown in Figure 2, a formulation consisting of 2 mM Cu^+^/10 mM Na Asc applied to unbleached cotton results in significantly enhanced levels of hydrogen peroxide compared with those found with the application of micromolar concentrations of Cu^+^/Asc. However, as shown in Figure 3, increasing the concentration of cotton-adsorbed formulations results in a corresponding increase in hydrogen peroxide generation that transitions from low-level generation to a level that borders on bacteriostatic activity (36 micromolar) (Hyslop,1995). Thus, as shown in Table 2, 2.5–3.2 mg/g of copper adsorbed on the cotton fabric in combination with millimolar applications of ascorbate or ascorbic acid results in H_2_O_2_ generation (5–30 micromolar hydrogen peroxide generation) over a twenty four-hour period. On the other hand, in situ formation of copper nanoparticles based on Cu^+^/Asc resulted in an increase of 20 micromolar hydrogen peroxide generation. Quantification of copper nanoparticle attachment responsible for this level of hydrogen peroxide, as shown in Table 2, was found to be 4 mg/g of fabric. It is notable that the singular application of 10 millimolar ascorbic acid to the unbleached cotton fabric results in a relatively higher production of hydrogen peroxide, i.e., 10–65 micromolar over an eighteen-hour period. This observation is consistent with previous findings on the relative roles of trace levels of copper in combination with ascorbic acid and other transition metal ions that mediate hydrogen peroxide formation, as well as the concentration effect of ascorbic acid as a pro-oxidant or an antioxidant functionality, i.e., what has been termed the cross-over effect [26,27].

In Figure 3, the hydrogen peroxide generation results of parallel evaluations of adsorbed Cu/Asc versus in situ synthesized Cu/Asc nanoparticles are shown.

Similar copper concentrations were applied to the fabric by way of adsorption and in situ formation, and the relative effect of sodium ascorbate versus ascorbic acid was evaluated for both adsorbed reagents and in situ synthesized nanoparticles. With adsorbed Cu/Asc, there is a stepwise increase in hydrogen peroxide production over a 24 h period. However, consistent with the results shown in Figure 2, singular applications of ascorbate or ascorbic acid produced a similar effect. On the other hand, in situ synthesis of copper nanoparticles with ascorbate versus ascorbic acid at different concentrations demonstrated differences in the efficiency of hydrogen peroxide production. For example, an optimal level of hydrogen peroxide production is observed with an increased amount of copper nanoparticles on the fabric (14.6 mg/g fabric), wherein 5 mM copper and 125 mM sodium ascorbate were employed for the in situ synthesis.

### 2.4. Antibacterial Activity and Assessment of Non-Leaching Adherence of Copper Complexes to Cotton

The results of the antibacterial activity of both sets of Cu/Asc analogs are shown in Table 3. A quantitative method for textiles was utilized, and the analogs were challenged with two bacterial strains. Effectiveness was determined after twenty-four hours of contact. The copper formulations prepared in this study ranged from 2.5–4.0 mg/g (see Table 1) cotton and all caused a ninety nine percent reduction of *S. aureus* and *K. pneumoniae*. 

**Figure 4:**
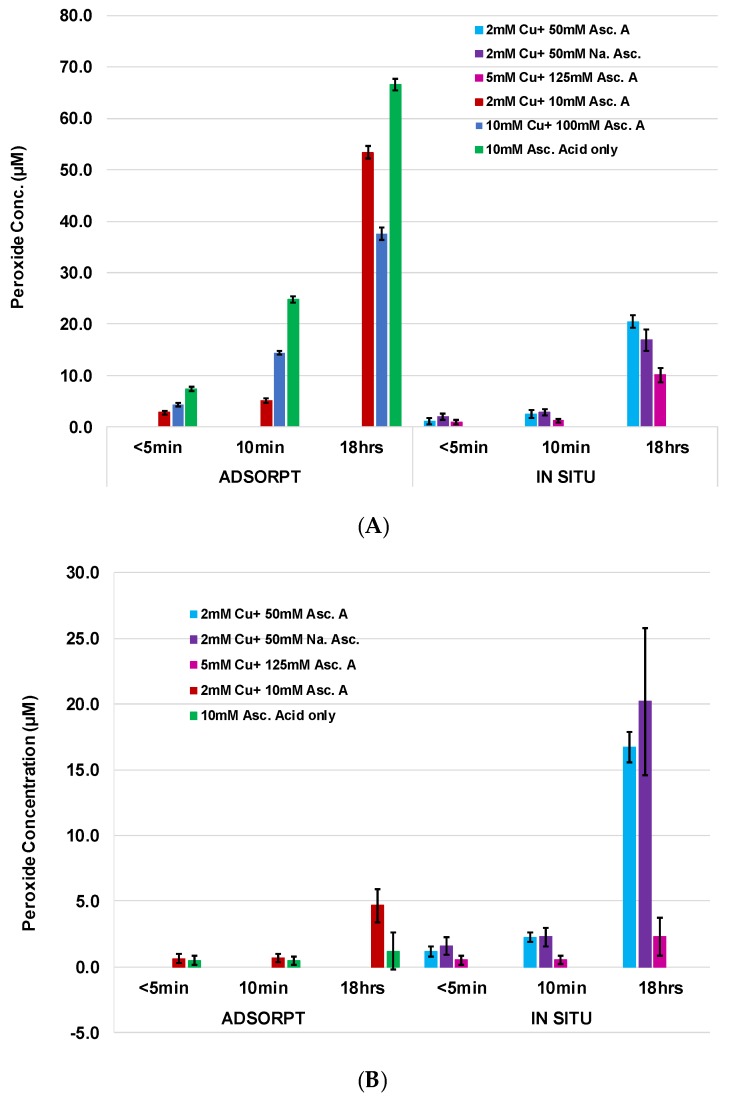
Peroxide generation of HE nonwoven greige cotton fabric determined by the Amplex Red assay, of adsorbed and in situ method treated samples (**A**) before and (**B**) after submersion in phosphate buffer pH 7.4 and blotted before assay, with the [AR] and [HRP] final concentration totaling 200 µM and 0.05 U/mL, respectively. Note: Untreated generated ~1.2 µM of peroxide.

The application of ascorbic acid to cotton demonstrates equivalent antibacterial activity to that found with the copper/ascorbate analogs. This is consistent with a previous report demonstrating the generation of hydrogen peroxide from ascorbic acid in the presence of trace amounts of copper and oxygen [28], and as Zhou et al. demonstrate, the reaction is strongly dependent on oxygen. This result is also consistent with the high peroxide levels observed with this formulation, as shown in Figure 4A. It also noteworthy that copper in its reduced form (Cu^+^) participates in a Fenton-like reaction (Equation (1)), where copper and hydrogen peroxide produce hydroxyl radicals and Cu^2+^. We have previously noted that this type of reaction may occur in the cotton fiber as a cyclical process of hydrogen peroxide and hydroxyl radical generation, which may enable prolonged coupling of the free radical hydroxyl product to hydrogen peroxide conversion and vice versa, the conversion of hydroxyl radicals by way of superoxide dismutase (SOD) dismutation, pectin hydrolysis, or polyphenolic autoxidation, which is found in the cotton fiber, to hydrogen peroxide [15,16,18].

Cu^+^ + H_2_O_2_ → OH + OH^−^ + Cu^2+^ (1)

To assess the copper/cotton materials as potentially durable and non-leaching, an evaluation of the level of hydrogen peroxide activity wash-out is important. Figure 4 compares the hydrogen peroxide activity of adsorbed Cu/Asc A with in situ synthesized nanoparticles following a water-only laundering. Activities prior to the leaching experiment are shown in Figure 4A. The activity of hydrogen peroxide decreased five-fold in the copper-adsorbed cotton formulation (3 mg/g). On the other hand, retention of activity was observed with the copper-in situ, cotton formulation.

**Figure 5:**
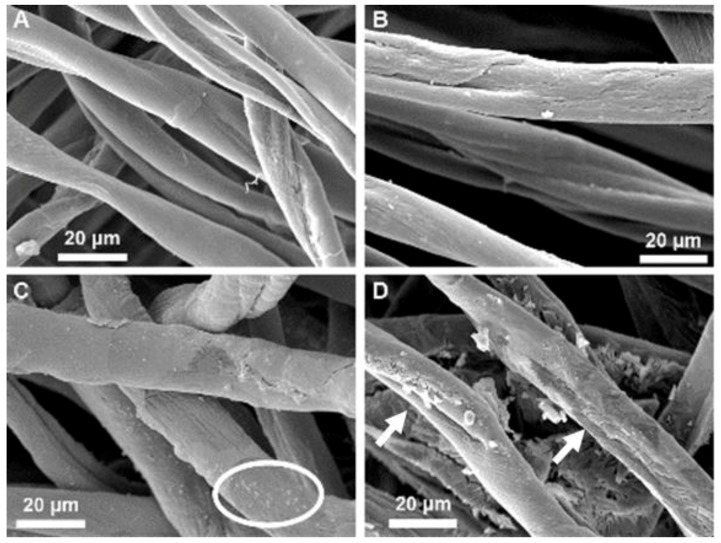
SEM images of (**A**) Untreated cotton nonwoven fabric; (**B**) Nonwoven fabric with adsorbed copper chloride; (**C**) Nonwoven fabric with copper nanoparticles; (**D**) Nonwoven fabric following ascorbic acid treatment. All images are shown at a 1200× magnification and with a 20 µm scale bar. The white circle in (**C**) indicates an area with Cu particles embedded in the fiber cuticle. Arrows in (**D**) point to surface damage following treatment with ascorbic acid.

As shown in Figure 5, SEMs of the Cu/Asc cotton analogs reveal that both the copper nanoparticles and microparticles are evenly dispersed on the unbleached cotton fibers. However, the copper nanoparticles appear embedded in the fiber cuticle and may even penetrate below into the primary and secondary cell wall. Whereas, the copper chloride results in less evenly distributed particles of varying size. Treatment and drying in the presence of ascorbic acid may also affect fiber morphology since the coated fiber surface appears rougher than the untreated one. Treatment with ascorbic acid alone reveals that it tends to coat the fiber cuticle as aggregated deposits that range from 5–20 micron particles. Thus, contact with the cuticle waxes upon slow drying results in precipitated crystalline ascorbate aggregates on the surface of the fiber.

### 2.5. Assessment of the Effect of Formulations on Fibroblasts and Implications for Design of Dressings

**Figure 6:**
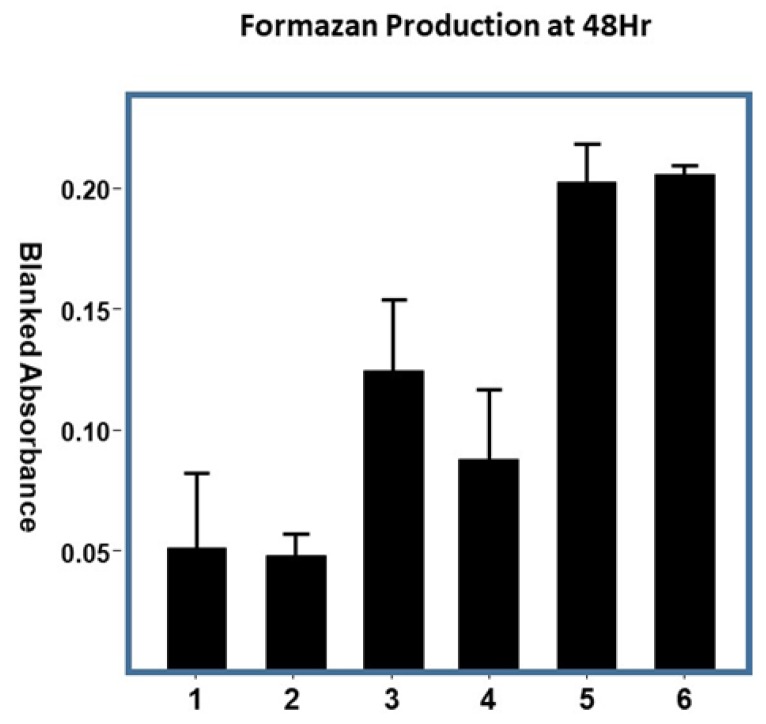
Cell Viability. Cell viability in response to the treatments was assessed after 48 h. exposure by measuring the reduction of a tetrazolium salt to formazan (*n* = 4). Samples of 8 mm diameter cotton disks were treated with the designated solution of copper nanoparticles: Sample (1) Greige Cotton + 100 mM Asc. Acid (Add on per weight of cotton: 162 g/mg); (2) Greige Cotton + CuNP + AA (Add-on per weight of cotton 609 g/mg); (3) Greige Cotton + 2 mM CuCl_2_ + 10 mM Asc (Add on per weight of cotton: 11 g/mg cotton). Acid; (4) Greige Cotton + 100 mM CuCl_2_ + 1 M Asc. Acid (Add on per weight of cotton: 515 g/mg; (5) 100 percent greige cotton only; (6) medium only. Error bars represent ± 1 standard deviation.

To assess the effect of Cu/Asc—analogs of cotton on fibroblast growth, direct treatment of human dermal fibroblasts was accomplished by incubation with cotton analogs and cell growth was determined by assessing the total fibroblast cell protein in parallel with hydrogen peroxide generation. As seen in Figure 6, the copper treatment attenuated the growth of fibroblasts grown in vitro in both the case of adsorbed analogs and nanoparticle annealed analogs. On the other hand, untreated cotton did not demonstrate evidence of attenuated growth.

## 3. Discussion

It is important to note that the in situ synthesis of copper nanoparticles on cotton was employed to develop non-leaching copper complexes annealed to the cotton fiber. A suitable approach for this type of modification has been adapted that utilizes green non-toxic reagents as ascorbic acid to reduce the copper salt to a nanoparticle [22]. Alternatively, copper nanoparticles embedded in the surface of fibrous paper (cellulose) may be reduced by “capping” agents like hydrazine or sodium borohydride, after making the cellulose alkaline with a base followed by reduction with ascorbic acid [23]. Notably, in an elegant approach to similar chemistry on cotton, it has been demonstrated that internally dispersed uniform silver nanoparticles form in the cotton fiber using ascorbic acid to reduce the silver without a stabilizing agent [29].

When greige cotton is hydroentangled into a nonwoven fabric, the secondary cell wall is exposed to improve absorbency, yet much of the components of the cotton fiber cuticle are retained [30]. This study examined greige cotton-based formulations as an approach to enhance and modulate peroxide production in wounds [31]. These unbleached cotton nonwovens, which retain the absorption capacity of bleached cotton and exhibit added functionality, have potential as an economical naturally occurring material for use in chronic wounds. This study examined the relative roles of copper and ascorbate to modulate hydrogen peroxide production when attached to greige cotton.

Previously, the qualitative pro-oxidant functionality of copper/ascorbate in the cotton fiber was demonstrated by inference based on ascorbate supplementation in the presence of trace levels of copper found in the cotton fiber [15]. To determine the stoichiometric relation of copper/ascorbate to hydrogen peroxide generation, a series of copper and ascorbate formulation concentrations was employed to pinpoint formulation ratios applicable to unbleached cotton which give hydrogen peroxide generation at a micromolar to millimolar threshold associated with cell proliferation or antibacterial activity, respectively [15,32,33]. In the previously cited study, it was shown that trace levels of copper present in the primary cell wall of the fiber are sufficient to yield hydrogen peroxide generation when ascorbate is adsorbed on the cotton fiber. However, based on a determination of the relative roles of copper and ascorbate in cotton and other plant tissue reported [16], supplementation of the cotton fiber with both copper and ascorbate was designed to produce hydrogen peroxide levels by adsorption on the nonwoven material within a micromolar to millimolar range. Also, it is important to note that hydrogen peroxide production results from oxygen-dependent oxidation of ascorbic acid via a copper redox-catalyzed reaction [28]. Moreover, the relevance of oxygen in hydrogen peroxide generation is viewed as critical in light of hypoxic chronic wounds, which is also pH dependent in the wound environment [34].

Previous work on the antimicrobial activity of copper and ascorbic acid is important to note in light of the findings of this work. Studies on the antibacterial effect of copper nanoparticles on cotton and cellulose report activity with different methods and degrees of efficacy, and found a generally higher efficacy against gram positive bacteria [35,36,37]. Multiple groups have suggested different modes of action attributable to copper nanoparticles. However, the generation of reactive oxygen species that in turn lead to lipid peroxidation, protein oxidation, and DNA degradation, is likely the principle antibacterial mechanism [38]. However, it is important to note that although antibacterial effects of copper and hydrogen peroxide have been discerned for their respective antimicrobial mechanisms of action, this study does not provide confirmation that H_2_O_2_ production was singularly required for hindering bacterial growth under the studied conditions. As discussed above, ascorbic acid produces some antibacterial effects both alone and in combination with other organic compounds and metals, and the concentrations of ascorbic acid applied to cotton in this study are below levels thought to exert a complete reduction of bacterial growth, as shown in Table 3. Verghese et al. reported a dose dependent inhibition of *K. pneumoniae* and *E. coli* with ascorbic acid solutions from 5–20 mg/mL [39]. However, in solution, these doses are only associated with the partial lowering of bacterial counts. Thus, it may be expected that some antibacterial effect on *K. pneumoniae* is expected from 5–10 mg/mL, which is two-fold more concentrated than the range of concentrations applied to the fabrics of this study since the add-on levels are approximately 2 mg/g cotton. However, the complete reduction of bacterial growth as shown in this study would not occur at this concentration with ascorbic acid alone. Thus, it seems likely that the ascorbic acid reacts with the trace levels of copper in the greige cotton fabric to produce bacteriostatic levels of hydrogen peroxide that inhibits bacterial growth at the ninety-nine percent level.

The non-leaching behavior of copper nanoparticles observed is consistent with previous reports that have discussed the application of copper nanoparticles to cellulose and cotton. For example, Sedighi et al. utilized a procedure previously reported for preparing permanent press fabrics on bleached and scoured cotton fabric [40] to anneal copper nanoparticles [35]. Copper sulfate was stabilized and reduced with citric acid and sodium hypophosphite, respectively, and repeated washing of the copper-cotton fabric showed no significant effect on the reduction of antibacterial activity. Eremenko et al. [37] also showed that the incorporation of bimetallic Ag/Cu on cotton is resistant to leaching and retention of antibacterial activity was observed.

The molecular basis for the stability of these types of metal nanoparticle-cellulose analogs has not been well characterized. However, it is thought that cellulosic hydroxyl oxygen lone pair electrons donate to the empty 4s, 4p, or d shell orbital of metallic copper to form a copper complex with four coordinate bonds [41], and basic conditions strengthen the negative charge coordination to copper (up to four coordinate bonds to hydroxyl oxygens). Complexation with ascorbate hydroxyls and carbonyl groups is also expected under the conditions of the nanoparticle formation reaction. In addition, copper nanoparticles may penetrate the matrix of the cotton fiber’s layers and microporous spaces, as has been shown with silver nanoparticle synthesis on cotton [29]. In this regard, Nam et al. showed that silver nanoparticles were uniformly formed throughout the entire volume of cotton fiber (edge and center). Since the diameter of cotton fiber is about 20 microns, the depth of the particle formation is considered to be the length to the core of the fiber around 10 microns. On the other hand, Yuranova et al. [42] reported a penetration of 30 a when silver and silver-titanium oxide particles were applied to bleached cotton. Thus, it is understandable that transition metal nanoparticles may form stable coordination complexes with cellulose and penetrate the cotton, enabling resistance to leaching. This, in part, may also account for some of the retention of activity of the adsorbed copper chloride (CuCl_2_) observed in this study (Figure 5B) since two of the coordination shells would bond to cellulosic hydroxyls.

Low-level hydrogen peroxide generation is essential for dermal wound healing, which is under redox control by way of NADPH oxidase, and is central to cell signaling events in the wound healing process [3,7,43]. Hydrogen peroxide has also been shown to promote re-epithelialization as an essential step in wound closure [44]. Furthermore, reactive oxygen species (ROS) and nicotinamide adenine dinucleotide phosphate (NADPH) oxidases promote the migration and proliferation of cells required for the repair of epithelial tissues and blood vessels [45]. Wound angiogenesis is stimulated by a low concentration of H_2_O_2_ and inhibited by catalase [3]. Low concentrations of H_2_O_2_ at the wound site can rescue repair in NADPH oxidase-deficient mice [4].

The effect of copper as cytotoxic to fibroblasts as shown in Figure 6 is not unexpected as cytotoxicity from copper to different types of fibroblasts in both normal and diseased patients has been reported previously [46,47,48]. Both positive and negative effects have been associated with the direct exposure of fibroblasts to hydrogen peroxide on human fibroblasts. For example, it has been shown that fibroblasts treated with hydrogen peroxide stimulate human melanoblast proliferation and melanocyte differentiation [49]. Levels of hydrogen peroxide similar to cell signaling have also been implicated in stimulating apoptosis (programed cell death), which in normal balance, is essential to healing [44,50]. On the other hand, a number of recent model studies show that fibroblasts resistant to hydrogen peroxide toxicity may be developed by using co-cultures of keratinocytes and fibroblasts and zebrafish as a model to definitively demonstrate positive effects of hydrogen peroxide on wound healing [44,51]. It also important to note that hydrogen peroxide-enhanced plasma-induced effluent, as demonstrated for infection and contamination mitigation, has been shown to affect fibroblasts and keratinocytes much less than bacteria [52]. The positive effects of low-level hydrogen peroxide are well documented and future work will evaluate these analogs with in vitro co-cultures to assess their potential to promote re-epithelialization [44]. Since numerous reports have documented the role of hydrogen peroxide in cell signaling and cell proliferation, the effects observed here are thought to be due to too high a level of copper and/or hydrogen peroxide altering cell growth. Due to the sensitivity of fibroblasts in vitro to hydrogen peroxide, it is appropriate to develop improved models that assess cell signaling levels of hydrogen peroxide. Thus, future studies will focus on evaluating analogs that stimulate low levels of hydrogen peroxide without a cytotoxic effect and developing improved in vitro models.

## 4. Materials and Methods

The hydroentangled nonwoven greige cotton (*Gossypium hirsutum* L.) fabric referred to as B9S-2 was a mixture of standard upland white cotton cultivars grown and harvested (2009–2010) in MS. The fibers were mechanically cleaned by T. J. Beall Co. (West Point, MS, USA), and distributed as True cotton© (TC©). True cotton© (TC©) was hydroentangled by the SRRC facility with the density of 38.9 g/m^2^. Horseradish peroxidase type I was purchased from Sigma Aldrich (St. Louis, MO, USA). Amplex Red reagent and Amplex Red^®^ Hydrogen Peroxide/Peroxidase Assay Kit cat # A22188 were purchased from Life Technologies (Invitrogen/Molecular Probes, Eugene, OR, USA). A novel tetrazolium compound, 3-(4,5-dimethylthiazol-2-yl)-5-(3-carboxymethoxyphenyl)-2-(4-sulfophenyl)-2H-tetrazolium, and inner salt (MTS), were purchased from Promega (Madison, WI, USA). All other chemicals were of a commercial reagent grade and used without further purification. 

Briefly, each fabric sample swatch, B9 S-2, was submerged and saturated with a solution of an appropriate concentration of copper (II) chloride hydrate and either l-ascorbic acid (Asc. A.) or l-sodium ascorbate (Na Asc.), including corresponding separate solution controls of CuCl_2_, ascorbic acid, and/or sodium ascorbate. Excess solution was removed by blotting and dried without tension in an 85 °C force draft oven for 15 min. They were allowed to equilibrate in air overnight before weighing and testing.

All samples assayed were cut into 8 mm circles and placed in 48-well plates or 5 mm circles in 96-well plates.

Variations to the general synthesis of copper nanoparticles inside cotton fiber were prepared to observe its effect on the size and species of copper particle formed. For in situ formation, the fabric was pretreated with 15% sodium hydroxide to swell the fibers. Excess base was removed by submersion in pure water and removing excess liquid. Sequentially, a solution of appropriate concentration of copper (II) chloride dissolved in pure water, Millipore 18 Ω, was applied to the fabric with the small addition of 2–3 M aqueous ammonia to adjust pH 10–12 soaking overnight. After blotting excess, the ascorbic acid or sodium ascorbate was added in an appropriate concentration ratio of 1:25, to reduce the copper and stabilize it.

An immediate yellow color forms and depending on the time allowed and temperature, pH, and heat added, the color turns red. The fabric was allowed to soak in the acid or salt solution overnight. The samples were rinsed in deionized water and dried in an 85 °C force draft oven for 10 min and then allowed to equilibrate overnight before testing. Depending on the concentration of copper, some of the dry fabrics’ color after equilibration to room conditions turned black and pale grey. 

The UV-VIS % reflectance spectra of the fabric swatches were measured in the wavelength range of 300–1000 nm using a UV2600 Shimadzu UV-VIS spectrophotometer utilizing the ISR-2600Plus integrating sphere attachment. The spectra were converted to % absorbance. 

The Amplex Red (AR) assay was used to measure peroxide generation/concentration, as previously published [15]. Briefly, 8mm circles in quadruplicate were placed in a 48-well microtiter plate, adding 200 µL of phosphate buffer (50 mM, pH 7.4) and 200 µL of Amplex Red(AR) (400 µM). Two hundred microliters of hydrogen peroxide standards, ranging from 20 µM to 0, and control solutions, at the same concentrations as the treatment solutions, were added separately to wells in duplicate and triplicate to the plate. To start the reaction, 10 µL of horseradish peroxidase (2 U/mL) was added to each well, with a final assay volume of 410 µL. The reaction was monitored fluorometrically detecting emission at 590 nm/30 of resorufin using the microplate reader Synergy-HT exciting at 530/30 nm. Background fluorescence, determined from zero concentration of standard, was subtracted from each value collected.

The AATCC Test Method 100 was performed by Situ Biosciences LLC (Wheeling IL 60090) [53]. Firstly, 48 mm diameter disks of the treated nonwoven samples were challenged with *Staphylococcus aureus* (4352) or *Klebsiella pneumoniae* (6538) by incubating the microorganism inoculum in contact with the test sample for a duration of up to 24 h without drying. Following exposure, the inoculated microorganisms were recovered and the concentration of the organisms was determined. The antimicrobial performance was determined by a comparison of the recovered organisms from the test samples at time 0, and treated material after selected time points was reported as a percent value relative to the control sample material.

The fabric samples, ~0.5 g, were digested with 28% nitric acid in water using CEM Mars 6 microwave one touch preprogram method filter-cellulose. The resulting solutions were diluted 4× and sent to Midwest Laboratories (Omaha, NE, USA) for total copper determination by inductively coupled plasma—atomic emission spectrometry (ICP-AES) using EPA method 200.7. The reporting limit was 0.01 in mg/L

To the cotton materials (2 mg), a total of 1 mL of Dulbecco’s Modified Eagle’s Medium (DMEM) without bovine serum, was added. Initially, 0.5 mL of medium was added to the samples and allowed to sit for a few minutes after vortexing. An additional 0.5 mL of medium was then added, the mixtures were vortexed for 2 min, and were finally sonicated for 10 min. The samples were then incubated in the dark overnight. Samples were centrifuged at 20,000 *g* at 4 °C for 15 min. Serum was added to 0.5 mL of the supernatant, to a final concentration of 10%. In triplicate, 100 µL of the serum containing supernatant was added to confluent fibroblasts in a 96-well microplate. The plates were incubated overnight in a CO_2_ incubator. Promega MTS reagent (20 µL) was added to the treated fibroblasts. An initial optical density (O.D.) was measured at 495 nm, the plate was incubated for 30 min, and the O.D. was measured again at 495 nm. To calculate the formazan produced, the initial O.D. for each well was subtracted from its final O.D.

The surface morphology of nonwoven fabrics was studied with the Philips XL 30 environmental scanning electron microscope (ESEM; FEI, Hillsboro, OR, USA). Fabric samples were mounted on an ESEM stub and coated with a gold/palladium coat. Each sample was examined using a 4.4 spot size, an acceleration voltage of 12 kV, and a working distance of approximately 6.5 m. Two magnifications were utilized to study the untreated fabrics, as well as those with additional materials, 500× and 1200×.

## 5. Conclusions

This study examines the relative roles of copper and ascorbate to produce hydrogen peroxide when attached to greige cotton for wound healing and antimicrobial applications and evaluates the sustained release of hydrogen peroxide production in a range associated with influencing cell signaling or antimicrobial effects to promote healing.

Historically, many commercial dressing materials have addressed the physical aspects of wounds, e.g., acting as a physical barrier to further injury, providing an optimal level of moisture, and removing excess exudate [54,55]. However, in recent years, focus on mechanism-based wound dressing research has addressed wound healing science concepts with the goal of incorporating functionality into dressings and biomaterials that enables the promotion of or re-entry into the major phases of wound healing [56,57]. Integration of functional design features in dressings has addressed growth factor delivery, protease modulation, antimicrobial activity, inhibition of biofilms, and prevention of scar formation, which enable the proliferative, inflammatory, and remodeling phases of wound healing. Low-level hydrogen peroxide generation (5–50 µM) has also been a subject of therapeutic interest for some time with regard to the promotion of cell proliferation Thus, developing an improved understanding of how wound dressings may be designed to address critical unsolved issues in wound repair and treatment is an impetus for the development of safe, economical, and highly functional materials for patients. Toward that end, we have characterized a series of formulations based on the inherent properties of the cotton fiber to produce hydrogen peroxide with an ultimate goal of understanding how hydrogen peroxide levels may be modulated for stimulating healing and antibacterial effects. The materials of this study demonstrated antibacterial effects, but the attenuated growth of fibroblasts suggests the importance of developing improved analogs that modulate hydrogen peroxide levels and assessing them in appropriate vitro models. It also raises considerations about discerning the overlap between bacteriostatic levels of hydrogen peroxide and fibroblast toxicity when developing treated wound dressing material, and the relative effects. Although numerous reports have been made on developing improved models to assess the cell signaling effects of hydrogen peroxide, few studies have sorted through the complex cellular effects in the wound that may occur with regard to exogenous agents on cell death and cell proliferation versus antibacterial effects. Future studies will focus on developing systems to examine the relative cytotoxicity versus enhanced cell proliferation so these types of formulations can be made tunable for incorporation into cotton-based dressing materials.

## Figures and Tables

**Table 1:** Total copper concentration of cotton samples determined by inductively coupled plasma-atomic emission spectrometry ^a^ (ICP-AES).

Sample Description	Digested Fabric Copper (Total) mg/L	Calc. Total Copper Per Fabric (mg/g)	% Add-On ^b^
5 mM CuCl_2_·2H_2_O + 125 mM Asc. Acid	190.8	3.31	-
2 mM CuCl_2_·2H_2_O + 125 mM Asc. Acid	82.4	1.51	0.25
2 mM CuCl_2_·2H_2_O + 50 mM Na. Asc.	225.2	4.20	0.52
5 mM CuCl_2_·2H_2_O + 125 mM Na. Asc.	756	14.64	0.75
2 mM CuCl_2_·2H_2_O + 10 mM Na. Asc.	170	3.23	1.2
2 mM CuCl_2_·2H_2_O + 10 mM Asc. Acid	152.8	2.53	0.96
Cotton ^c^ control	0.52	0.01	
Untreated brown cotton ^d^	0.72	0.01	

^a^ Copper total was determined after microwave acid digestion by ICP-AES according to EPA method 200.7 and reported in mg/L; ^b^ Add-on percent calculated for samples 1–4 after rinsing fabric and 24 h equilibration; samples 5–6 add-on percent calculated after 24 h equilibration; ^c^ Untreated hydroentangled upland white cotton (unbleached); ^d^ Untreated brown cotton variety B-14.

**Table 2:** Peroxide Production of copper (II) chloride in sodium ascorbate solution measured amplex red assay ^a^.

Concentration of Solution	Peroxide Concentration (µM)				
Components	0 h	0.5 h	2 h	3 h	24 h
**200 µM Na Ascorbate**					
20 µM Cu	13.43	26.80	26.33	26.27	29.06
2 µM Cu	19.54	30.64	29.86	29.70	32.44
200 nM Cu	5.03	16.09	16.29	16.38	21.71
20 nM Cu	5.69	10.59	10.60	10.72	14.91
2 nM Cu	6.04	9.53	9.49	9.61	13.71
200 pM Cu	6.92	9.09	9.07	9.18	13.03
0 µM Cu-ascorbate only	6.45	8.02	7.95	8.10	11.40
**100 µM Na Ascorbate**					
20 µM Cu	16.10	23.70	23.30	23.22	25.02
2 µM Cu	18.98	25.45	25.00	24.91	27.83
200 nM Cu	2.08	2.67	3.21	3.61	10.62
20 nM Cu	0.83	0.85	0.99	1.09	4.38
2 nM Cu	0.58	0.58	0.64	0.69	3.35
200 pM Cu	0.52	0.54	0.58	0.63	3.09
0 µM Cu-ascorbate only	0.51	0.53	0.57	0.61	2.83
**20 µM Na Ascorbate**					
20 µM Cu	3.96	4.60	4.55	4.57	5.45
2 µM Cu	4.52	5.18	5.11	5.14	6.38
200 nM Cu	0.77	0.80	0.93	0.99	1.65
20 nM Cu	0.58	0.59	0.62	0.65	1.24
2 nM Cu	0.55	0.55	0.57	0.59	1.10
200 pM Cu	0.54	0.54	0.56	0.57	1.04
0 µM Cu-ascorbate only	0.51	0.52	0.53	0.54	0.96

^a^ The peroxide concentration is an average of triplicate determination with a calculated deviation of ± 0.5 µM.

**Table 3:** Results of Assessment of Formulations in AATCC Test Method 100 for quantitative assessment of antibacterial finishes on textile materials.

Sample Description	*K. Pneumoniae*	*S. Aureus*
	@ 24 h	
2 mM Copper only	99.98	99.99
2 mM Cu:10 mM Ascorbic Acid	99.98	99.99
10 mM Ascorbic Acid only	99.98	99.99
2 mM Cu:50 mM Ascorbic Acid (CuNP)	99.98	99.99
2 mM Cu:10 mM Sodium ascorbate	99.98	99.99
2 mM Cu:50 mM Sodium ascorbate (CuNP)	99.98	99.99
Untreated Control	0	0
SBSC Untreated Control	9.54 × 10^6^ CFU/mL	7.4 × 10^4^ CFU/mL

Inoclum *K. pneumoniae* bacterial concentration at 0 h 2.3 × 10^5^ CFU/mL and at 24 h 9.54 × 10^6^ CFU/mL. *S. aureus* concentration at 0 h 3.5 × 10^5^ CFU/mL and at 24 h 7.4 × 10^4^ CFU/mL Percent reduction at 24 hours as defined in the Materials and Methods section.
